# Addressing poverty post COVID-19 pandemic

**DOI:** 10.1016/j.lanepe.2021.100156

**Published:** 2021-06-07

As per data from the UN's Human Rights Agency, one in five people, or 21.1% of the EU's population (EU-27), were at risk of poverty or social exclusion in 2019, representing 92.4 million people. A total of 19.4 million children, representing 23.1% of all EU children, are at risk of poverty across the Union. Women in particular experience higher poverty rates than men, and this gap only grows wider with old age due to inadequate pensions, for which the average gender gap is a staggering 37.2% across the EU. The EU's commitment—Europe 2020 target—to lift 20 million people out of poverty by 2020 has been missed, despite steady economic and employment growth before the Covid-19 pandemic. The pandemic has disproportionately impacted the poorest and affected many Europeans who had never confronted poverty before, inflating the inequality gap and poverty numbers—the exact extent of which will take years to be determined. COVID-19 poses a real challenge to the UN Sustainable Development Goal of ending poverty by 2030, and it is estimated that the poverty headcount rates could increase for the first time since mid-1990s in Europe.

One of the three commitments taken by the EU leaders, European institutions, social partners, and civil society representatives at the Porto Social Summit held on May 7, 2021, was to “reduce the number of people at risk of poverty or social exclusion by at least 15 million, including at least 5 million children by 2030”. These targets are encouraging, but if the 2020 targets were not met, what is the hope that 2030 targets will be? There is no clarity on what lessons EU institutions have drawn from the failure to meet the Europe 2020 target, and what could be done differently in the future.

Although having ambitious targets and identification of poverty figures is crucial, there are fundamental problems in the way poverty is monitored and dealt with, and the COVID-19 pandemic has exposed these deficiencies across the EU. First, there is a huge disparity in the way poverty is calculated, both between countries and within a country. The official poverty line needs to be determined, the definition of poverty needs to be better defined, and the choice of poverty indicators—that are largely politically determined—needs to be expanded and made more inclusive beyond absolute and relative measures. For example, digital deprivation should be included as one of the indicators that has become obvious during the pandemic. Other factors such as geography, local vulnerabilities, health poverty, and health-care access for marginalised communities need to be considered.

Second, the gaps in poverty-monitoring capacity since the past decade have increased during the pandemic. Poverty data are typically drawn from household surveys, which have been affected by the pandemic, and therefore the actual extent of poverty during the pandemic would be difficult to be accurately determined. There is a shift from direct estimates from surveys to model-based estimates, and data integration resources of member states are very scarce—which makes accurate and inclusive poverty monitoring a challenge. Moreover, appropriate standards need to be set to determine who should be included in the measurement—homeless people are frequently excluded from data collection, and households without phone or internet access would be missed.

Third, long-term support and financial aid are required, specifically for the most vulnerable sections of society—the so-called new poor, primarily comprising people without digital access. The pandemic has shifted the workforce dynamics towards reliance on automation and artificial intelligence, which has been a technological advancement but, at the same time, decreased the low-skilled labour demand and led to obvious job cuts—increasing the inequality between the digital and non-digital class and adding a push towards poverty. This pandemic-induced inequality is also more evident when compared with that of the 2007 Global Financial Crisis, because the nature of the present health crisis has prevented jobs in high-contact sectors that are done usually by low-skilled people. This new imbalance needs to be bridged by active labour market policies that would provide opportunities to the new poor to join the active workforce and escape the poverty trap.

Fourth, specific and long-term health-care protection and investment needs to be allocated for poor individuals and particularly for every child at risk of poverty. Children from poor households, who did not have access to digital methods of education during the pandemic, have been most severely affected. Without effective help, the risk of poverty is passed on from one generation to the next. Young people who grow up in poverty are generally more vulnerable and more likely to be in poor health and malnourished, to have learning and behavioural difficulties, to underachieve at school, to have lower skills and aspirations, to have low-paid jobs, and to be unemployed and dependent on welfare. Physical and psychological effects of deprivation are likely to deepen poverty.

To avoid repeating the same mistakes of the past, there is a need for EU-wide strategy to move beyond merely setting targets towards implementing coherent measurements and strong and nuanced policies to counter the adverse effects of COVID-19 on poverty and the widening inequality in society. Fiscal and other macro policies should be calibrated to achieve equitable and sustainable growth. Investments in health care, education, and social protection should be continued for the long term. Importantly, until economic recovery is assured, the fiscal support should not be withdrawn prematurely, as was the case after the Global Financial Crisis. In the absence of supportive policies to protect the vulnerable, the pandemic will widen the existing inequality gap and push even greater numbers into poverty.

*The Lancet Regional Health - Europe*

